# Plant-Based Functional Custard Enriched with Vitamin D_3_: Insight into Technological Properties, Nutrition and Bone Tissue Regeneration

**DOI:** 10.3390/foods15183337

**Published:** 2026-09-20

**Authors:** Marwa A. Sheir, Rania A. Ahmed, Francesco Serrapica, Abeer M.F. Elbaz

**Affiliations:** 1 Special Food and Nutrition Department, Food Technology Research Institute, Agricultural Research Center, Giza 12613, Egypt; 2 Department of Zoology, Faculty of Science, Suez University, Suez 43511, Egypt; rania.sheir@yahoo.com; 3 Department of Agricultural Sciences, University of Naples Federico II, 80055 Portici, Italy; 4 Food Engineering and Packaging Department, Food Technology Research Institute, Agricultural Research Center, Giza 12613, Egypt; drabeer_elbaz@yahoo.com

**Keywords:** bone repair, custard, plant-based diet, vegan, vitamin D_3_

## Abstract

A plant-based or vegan diet is considered one of the most important dietary patterns, often followed for religious or health reasons. It should contain vital nutrients such as protein, iron, zinc, calcium, vitamin D, and others. The objective of this study was to produce a plant-based food product with high nutritional value (vegan custard, VC) and fortify it with vitamin D_3_ (vegan custard + D_3_). Thirty-five female albino rats, with an average body weight of 160 ± 5 g, were divided equally into five groups (seven rats per group): Negative control group: Rats were fed only on a normal-calcium diet. Positive control group: Rats were fed only on a low-calcium diet. Group I: Low-calcium diet supplemented with 10% commercial custard; Group II: Rats were fed a low-calcium diet supplemented with 10% vegan custard; Group III: Rats were fed a low-calcium diet supplemented with 10%-D_3_-fortified vegan custard. The quality, nutritional values, and calcium bioaccessibility of the custard samples were compared with commercial custard. The effect of feeding rats all types of custard on body changes, as well as certain serum biochemical markers such as calcium, parathyroid hormone (PTH), calcitonin, tartrate-resistant acid phosphatase (TRACP), and alkaline phosphatase (ALP), was assessed. Additionally, femur calcium content and bone tissue improvement were histologically examined. Results showed that the innovative vegan custard samples (VC and VC+D_3_) achieved the highest scores in consumer palatability, nutritional value, and manufacturing properties. Notably, the D_3_-fortified vegan custard increased calcium bioaccessibility by up to 85.3% and can provide 333.3% of the daily vitamin D requirements per approximately 100 g. The product also provides 3.12–4.40% of the recommended daily calcium intake per 100 g, depending on age-specific requirements. Furthermore, histological investigations and histomorphometric evaluations revealed significant improvements in femoral bone tissue improvement. Finally, a daily serving of vegan custard prepared from 30 g of D_3_-fortified custard powder is recommended to meet vitamin D requirements.

## 1. Introduction

Custard powder is a useful textured dry food product used as breakfast cereal or weaning food in many developing countries, especially in the tropics. Basically, it is corn starch processed with added flavors and fortified with essential minerals and vitamins [1,2]. Custard paste or gruel is manufactured by mixing custard powder with water, followed by the addition of boiling water to achieve the desired consistency [1,3].

A plant-based or vegan diet consists mainly of plant foods such as nuts, vegetables, legumes, whole grains, seeds and fruit with little or no animal products. This diet pattern has become popular around the world due to concerns about human health, animal welfare and environmental sustainability. It has been reported in many studies to have potential health benefits such as reduced risk of chronic diseases such as type 2 diabetes, hypertension and some cancers such as colorectal, breast and gastric cancers. Other benefits are lower blood cholesterol levels and reduced risk of gallstones and intestinal problems [4,5]. However, vegans need to make sure that they are getting sufficient nutrients such as protein, zinc, iron, calcium, vitamin D, vitamin B12, and omega-3 fatty acids [6]. Of particular concern among these nutrients are vitamin D and calcium, given their important role in bone metabolism and the limited availability of naturally rich plant-based dietary sources.

Vitamin D is a fat-soluble vitamin that plays a key role in calcium balance and bone physiology. This vitamin is involved in immune function and glucose regulation. A low level of this vitamin may be responsible for the higher risk of various diseases such as diabetes mellitus, cancer, CVD, and multiple sclerosis [7,8]. Vitamin D comes in two varieties: ergocalciferol (vitamin D_2_) and cholecalciferol (vitamin D_3_); however, both have similar metabolic pathways. They are first hydroxylated in the liver to form 25-hydroxyvitamin D (25(OH) D), then further hydroxylated in the kidney to produce the biologically active form, 1,25-dihydroxyvitamin D (1,25(OH)_2_D). Yet, endogenously produced vitamin D_3_ may not be enough to fulfill the needs of the body because of various reasons such as aging, geographical position, skin coloration, and sun protection behaviors.

Previous studies have proven the efficacy of fortified foods with vitamin D. It has been shown that the deficiency of vitamin D can disrupt the signal pathways associated with the development and survival of cells [9]. The deficiency of vitamin D is highly prevalent all over the world, especially in the Eastern Mediterranean region [10]. Despite the controversy over the role of vitamin D in the health of adult bones, it is known that vitamin D is an important regulator of the balance between calcium and phosphorus, which is critical for the development and maintenance of healthy bones. Vitamin D improves the absorption of calcium in the intestine and promotes bone mineralization. The deficiency of vitamin D causes low efficiency of calcium absorption, resulting in poor bone mineral density and higher susceptibility to osteoporosis and bone fracture [11,12].

Although fortified food products are becoming increasingly available, functional vegan custard products enriched with vitamin D_3_ and designed to improve calcium intake remain limited. This highlights a clear gap in the development of fortified plant-based custard formulations. Custard powder represents a suitable vehicle for nutrient fortification due to its wide consumer acceptance, ease of preparation, and compatibility with functional ingredients without adversely affecting sensory quality [1,2].

In this case, the choice of proper plant-based ingredients with high nutritional and functional values plays an important role in developing fortified products. Pearl millet and baobab flour are ingredients that have high levels of minerals, especially calcium, along with functional properties [13,14]. Also, banana was added as a natural flavoring and as a source of important micronutrients, contributing to both the nutritional quality and sensory acceptability of the final product [15].

Accordingly, the present study aimed to produce a novel plant-based custard product naturally enriched with calcium and fortified with vitamin D_3_. The developed formulation was designed to provide high nutritional value to enhance calcium and vitamin D_3_ intake and may represent a suitable functional food option for vegetarians, individuals with dairy allergies or lactose intolerance, and populations at risk of micronutrient deficiencies.

## 2. Materials and Methods

### 2.1. Materials

#### 2.1.1. Source of Plant Materials

Corn starch (12% moisture), vanilla powder, sugar, and commercial custard (Cook’s) were obtained from Carrefour Market, Egypt. The commercial custard consisted of food-grade corn starch, vanillin, authorized artificial flavors, and authorized natural color (E101) and may contain traces of milk derivatives. Baobab flour (BF) was acquired in Aswan, Egypt. Pearl millet grain (Pennisetum americanum), var. Shandawel-1 was obtained from Field Crops Dep., Agr. Res. Center, Giza, Egypt, and ground to pass an 80-mesh sieve.

#### 2.1.2. Source of Other Materials

The kits, tartrazine (E102) and vitamin D_3_ (cholecalciferol) were obtained from Sigma-Aldrich (St. Louis, MO, USA). Other chemicals and solvents were obtained from Scharlab S.L. (Sentmenat, Barcelona, Spain). All chemicals and reagents used were of analytical grade.

### 2.2. Methods

#### 2.2.1. Preparation of Banana Powder

Fresh bananas were peeled and sliced into uniform slices (2.5 mm thickness). A fixed batch of banana slices was immersed in 600 ppm sodium metabisulfite solution for 5 min to prevent enzymatic browning [16]. The slices were then dried using vacuum-infrared drying at 0.8 bar and 80 °C [17]. The dried slices were ground into powder using a mixer, sieved through a 0.50 mm mesh, and packaged in polyethylene bags.

#### 2.2.2. Formulation and Preparation of Innovative Vegan Custard Samples

The vegan custard (VC) was prepared using corn starch (580 g), baobab flour (100 g), millet flour (70 g), banana powder (230 g), vanilla powder (19 g), and tartrazine (E102) at a level of 1 g/kg as a lemon-yellow colorant. For vitamin D_3_ fortification (VC+D_3_), the same formulation was used, with vitamin D_3_ added at a concentration of 25 μg per 50 g of the innovative vegan custard, as previously reported [18]. To produce the vitamin-D_3_-fortified vegan custard sample, vitamin D_3_ was added in powder form and gradually incorporated into the formulation. Homogenization was carried out using a high-speed water-cooled homogenizer (Janke & Kunkel K.G., Typ A10, Staufen i. Br., Germany) at a maximum speed of 20,000 rpm, with a power output of 75 W and an operating voltage of 220 V (50 Hz). To prevent overheating, homogenization was performed in short intervals, with a maximum duration of 5 min, in accordance with the manufacturer’s recommendations. This process ensured a more uniform distribution of vitamin D_3_ throughout the custard matrix.

#### 2.2.3. Preparation of Custard Desserts

For custard preparation, 25 g of each sample, including the commercial custard, was transferred into clean beakers. Subsequently, 40 g of sugar and 100 mL of tap water were added and thoroughly mixed. Then, 300 mL of boiling water was added to the commercial custard sample, strictly according to the preparation instructions recommended by the manufacturer and stated on the product label, while 150 mL of boiling water was added to each vegan custard sample. The water-to-powder ratio of the developed vegan custard was optimized during preliminary formulation trials to achieve the desired consistency. Therefore, each formulation was prepared according to its respective optimized preparation conditions. All samples were cooked in a water bath at 95 °C for 30 min and then allowed to cool to room temperature. The prepared custard desserts were subsequently transferred into containers and stored at 4 °C until further analyses.

#### 2.2.4. Quality, Nutritional and Functional Assessment of Custard Samples

##### Color

A color meter (Color Tec PCM™, Color Tec Association Inc., 28 Center Street, Clinton, NJ, USA) was used to measure the color parameters of custard powder and cooked custard samples. The colorimeter operates according to the Commission Internationale de l’Éclairage (CIE) L*, a*, b* color system. Multiple measurements were taken at different points on each sample. Prior to analysis, the instrument was standardized using a sheet of Business Xerox 80 g/m^2^ white paper with 36 CIE whiteness D65 values (L* = 93.24, a* = 0.96, b* = −2.75). Approximately 3 g of each sample was placed on a clean sheet of paper, and the color meter sensor was positioned directly in contact with the sample surface to obtain the readings. The instrument expresses color differences in three-dimensional uniform color space (Lab coordinates), representing variations from light to dark and from red to green or yellow to blue. Chroma (C*) was calculated according to the equation $C*=\sqrt{{a*}^{2}+{b*}^{2}}$, while hue angle (H°) was calculated as arctan (b*/a*).

##### Sensory Evaluation

Samples were assessed using a nine-point hedonic scale, based on appearance, flavor, mouth feel, taste and overall acceptability as described by Singh and David [19]. Twenty judges from the Food Technology Research Institute, Agricultural Research Center, Giza, Egypt, specialized in food research and evaluation, participated in the sensory assessment. Scores ranged from 1 (extremely dislike) to 9 (extremely like).

##### Solubility

Solubility was determined at 40, 50, and 60 °C according to the method described by Cano-Chauca et al. [20], with some modifications. Briefly, 100 mL of distilled water was transferred into a blender jar. A powder sample (1 g, dry basis) was carefully added and blended at high speed for 5 min. The resulting solution was transferred into centrifuge tubes and centrifuged at 3000× *g* for 5 min. An aliquot of 25 mL of the supernatant was transferred into pre-weighed Petri dishes and immediately oven-dried. Solubility (%) was then calculated based on the weight difference.

##### Bulk Density

For bulk density determination, 7 g of custard powder was placed into a 50 mL graduated measuring cylinder. A constant volume was obtained by gently tapping the cylinder against the palm of the hand. Bulk density was then calculated as the ratio between sample weight and sample volume. Lower bulk density may facilitate packaging and transportation efficiency by reducing the volume occupied by the fortified custard blend during storage and handling [21].

##### Dispersibility

Dispersibility was determined according to the method described by Kulkarni et al. [22], with some modifications. Briefly, 10 g of each sample was mixed with 100 mL of distilled water and stirred thoroughly. The mixture was then vigorously shaken and allowed to settle for 3 h. Dispersibility was calculated using the following equation:

%Dispersibility=100 mL−Volume of settled particles

##### Texture Profile of Cooked Custard

Texture profile analysis was performed using a texture analyzer (Model EZ-SX, Shimadzu Corporation, Kyoto, Japan) equipped with a cylindrical probe (50 mm diameter). The analysis was conducted at a compression distance of 10 mm, with pre-test and post-test waiting times of 5 s and a crosshead speed of 2 mm/s. The collected data were analyzed using the texture analyzer’s built-in software.

##### Evaluation of the Nutritional Value of Custard Samples

The custard samples were analyzed according to the standard methods described by Latimer [23] for the determination of moisture, crude protein, crude fiber, and crude fat contents on a dry weight basis. Available carbohydrates (dry basis) were calculated according to the following equation [24]:

%Available carbohydrates=100−(%Ash+%Fat+%Protein+%Fiber)

Energy value (kcal/100 g) was calculated according to James [25] using the following equation:

Energy (kcal)=[Protein (g)×4]+[Carbohydrate (g)×4]+[Fat (g)×9]

Mineral contents, including calcium (Ca) and phosphorus (P), were determined using an atomic absorption spectrophotometer (Model 3300, PerkinElmer, Waltham, MA, USA) [23]. Vitamin D content was quantified by high-performance liquid chromatography (HPLC) according to Bošnir et al. [26], whereas vitamin C content was determined spectrophotometrically [27].

##### Antioxidant Activity and Total Phenolic Content

The free radical scavenging activity of custard samples was evaluated using the DPPH assay [28]. Total phenolic content was determined spectrophotometrically using a modified Folin–Ciocalteu method and expressed as gallic acid equivalents (GAE) based on a gallic acid standard curve [29].

##### In Vitro Calcium Bioaccessibility

Calcium bioaccessibility of all samples was evaluated by in vitro enzymatic digestion according to the method described by Suliburska and Krejpcio [30] with some modification. Briefly, 2 g of each sample was mixed with 20 mL of deionized water in a conical flask. The pH of the mixture was adjusted to 2.0 using 0.1 N HCl, followed by the addition of pepsin (0.5 mL/100 mL homogenate).

Each sample was incubated in a thermostatic water bath at 37 °C for 2 h under continuous agitation while monitoring the pH. Subsequently, the pH was adjusted to 6.8–7.0, and pancreatin (10 mL/40 mL homogenate) was added. The samples were then incubated under the same conditions for an additional 4 h. After digestion, the mixtures were centrifuged at 3800 rpm for 10 min.

An aliquot of 10 mL of the supernatant was transferred into Teflon vessels, mixed with 5 mL of concentrated nitric acid (Merck, Darmstadt, Germany), and mineralized using a MARS-5 microwave digestion system (CEM, Matthews, NC, USA). Calcium concentration was then determined by atomic absorption spectrometry (AAS spectrometer-3, Zeiss, Oberkochen, Germany).

All analyses were performed in triplicate. Calcium recovery percentage was 105.6%, and method accuracy was verified using a certified reference material (Brown Bread BCR191, Sigma-Aldrich). Relative calcium bioaccessibility was expressed as the percentage ratio between the amount of calcium released after enzymatic digestion and the total calcium content present in the sample.

#### 2.3. Animals and Experiment Design

##### 2.3.1. Preparation of Animals’ Diet

The ingredients of the normal-calcium diet and low-calcium diet were prepared according to Chen et al. [31] (Table 1). The normal-calcium diet contained casein (24.5 g/100 g), corn oil (6.0 g/100 g), corn starch (37.5 g/100 g), potato starch (1.0 g/100 g), sucrose (15.5 g/100 g), cellulose (5.0 g/100 g), vitamin mix (1.0 g/100 g), mineral mix (7.0 g/100 g), and CaCO_3_ (2.5 g/100 g). The low-calcium diet had the same composition except that the corn starch and CaCO_3_ contents were adjusted to 39.87 g/100 g and 0.13 g/100 g, respectively.

##### 2.3.2. Animal Grouping

In the current study, thirty-five female Wistar albino rats weighing (160 ± 5 g), were purchased from Medical Analysis Department, Research Institute of Ophthalmology, Giza, Egypt, then were left for one week to acclimatize and to exclude any abnormal animal. All animals were housed under suitable environmental conditions (humidity 30–60%, temperature 22–24 °C, 12 h light/12 h darkness). Animals were allocated into five experimental groups (7 rats in each group). Each rat was housed individually in a separate cage throughout the experimental period. The negative control group was fed a normal-calcium diet for 30 days, while the positive control group was fed a low-calcium diet for the same period. Group I received 90% low-calcium basal diet +10% commercial custard for 30 days, whereas Group II received 90% low-calcium basal diet +10% vegan custard for 30 days. Group III received 90% low-calcium basal diet +10%-D_3_-fortified vegan custard for 30 days.

The animals were monitored daily throughout the study for general health status, behavior, and signs of distress. No unexpected morbidity or mortality was observed.

Every day, the amount of diet offered to each animal and the remaining and/or wasted diet were recorded, and individual daily feed intake (FI) was calculated. The individual rat was considered the experimental unit for feed-intake measurements. Daily nutrient intakes (protein, energy, calcium, and vitamin D_3_) were calculated from the mean daily feed intake and the nutrient composition of the corresponding diet. Additionally, body weight was recorded. Feed efficiency ratio (FER) and body weight gain percent (BWG %) were calculated [32], using the following equations:

BWG% = (Final body weight − Initial body weight)/Initial body weight × 100

FER = Weight Gain (g)/Feed intake (g)

##### 2.3.3. Histological Investigation

At the end of experiment, all animals were sacrificed by cervical dislocation by a trained investigator, in accordance with internationally accepted guidelines for the care and use of laboratory animals; and the left femurs were dissected out and cleaned. The dry weight of the femur was measured by a precision balance. Ca content in femurs was determined according to Wawrzyniak et al. [33].

Other femur bone samples were preserved for 24 h with 10% buffered formalin for histological examinations. Osteodec was then used to decalcify the specimens. Each specimen was then regularly treated before being individually embedded in paraffin blocks. Hematoxylin and eosin were used to stain the two-micrometer-thick sections that were cut from the blocks (three sections for each tissue sample). Two femoral bone slices were shown on each slide. Every slide was examined using a high-power field (HPF; 100× magnification) light microscope (Leica, Allendale, NJ, USA) [33]. Histological evaluation was performed using predefined standardized criteria to ensure consistency of assessment across all samples.

##### 2.3.4. Histomorphometric Quantitative Analysis

Histologic photomicrographs of the H&E-stained sections (all obtained at ×100 original magnification) were quantitatively analyzed for the regenerative process outcome using image analysis software (image J 1.46r software (24)). The osteoblastic count (OC) and cortical bone thickness (CBT) were measured for experimental and control groups. Each of OC and CBT was measured in five non-overlapping microscopic fields for each animal. The average of these five measurements was considered the final value for each animal. Statistical analysis was performed using these animal-level values (*n* = 7 animals per group).

##### 2.3.5. Biochemical Investigation

Blood samples were collected via the orbital sinus technique [34], allowed to clot, and centrifuged at 5000 rpm for 10 min. Sera were separated and stored at −20 °C. Serum calcium concentration was determined using a standard colorimetric method [33]. Serum parathyroid hormone (PTH) and calcitonin levels were measured using rat-specific immunoradiometric assay kits (Immutopics, Inc., San Clemente, CA, USA). Tartrate-resistant acid phosphatase (TRACP) activity was assessed according to the manufacturer’s instructions (ShanghaiYiyan Biotechnology Co., Ltd., Shanghai, China). Alkaline phosphatase (ALP) activity was determined according to Belfield and Goldberg [35].

#### 2.4. Statistical Analysis

All experiments were analyzed using a one-way ANOVA via SPSS software (version 22, IBM Corp., Armonk, NA, USA 2013). Data from each experimental assay were treated according to a completely randomized design with seven replications per group [36]. Multiple comparisons among treatment means were carried out using Duncan’s multiple range test, with statistical significance set at *p* < 0.05

### 3. Results and Discussion

#### 3.1. Color Parameters of Custard Samples

Color parameters in the CIE tristimulus color space (Lab) are presented in Table 2 for custard powder samples and in Table 3 for cooked custard samples. The commercial custard powder exhibited the highest lightness value (L* = 99.59), indicating an almost absolute white color, together with a negative a* value (−2.81) and a positive b* value (9.34), reflecting a yellow-greenish color tone. Correspondingly, the chroma value (C* = 9.76) was relatively low, while the hue angle reached 106.77°, confirming the yellow-greenish hue of the product. These findings are consistent with those reported by Zarroug et al. [37], who indicated that high L* values combined with relatively low chroma values are indicative of color purity. The yellow-greenish coloration observed in the commercial custard powder may be attributed to the added coloring agents, including tartrazine and sunset yellow.

Incorporation of banana, baobab, and millet flours into the corn starch formulation markedly altered the color characteristics of the vegan custard powders. In the vegan custard (VC), lightness decreased to 89.77, whereas redness (a*) and yellowness (b*) increased to 4.03 and 12.71, respectively. Consequently, chroma increased to 13.33, while the hue angle shifted toward an orange-yellow color tone (72.43°). Similar changes in the color properties of corn-starch-based formulations enriched with plant ingredients have previously been reported by Su et al. [38] and Zarroug et al. [37].

The addition of vitamin D_3_ did not noticeably affect lightness or yellowness values; however, the VC+D_3_ sample showed a higher redness value (a* = 4.85), resulting in a slightly more orange hue angle (68.78°). The color parameters of cooked custard samples are presented in Table 3. Cooking markedly reduced lightness values in all samples, reaching 66.46, 55.81, and 55.13 for commercial custard, VC, and VC+D_3_, respectively. This reduction may be attributed to heat-induced color development during cooking.

Both commercial and vegan cooked custard samples exhibited negative a* values, indicating slight greenish color tones, whereas the VC+D3 sample shifted toward positive a* values, reflecting a more reddish appearance. In addition, the yellowness values (b*) of Please confirm if the italics are necessary; if not, please remove them. 

Vegan custard samples increased substantially (B* = 66.16–66.18), likely due to the incorporation of plant-based ingredients. Correspondingly, the vegan cooked custard samples exhibited higher color saturation (C* = 66.18–66.26) compared with the commercial sample (C* = 25.26). Hue angles also shifted toward a more intense yellow hue (93.12–88.62°) relative to the yellow-greenish hue of the commercial custard (107.22°).

Overall, the incorporation of banana, baobab, and millet flours enhanced color saturation and produced more visually appealing custard products. These observations are in agreement with the finding of Zarroug et al. [37], who reported that enrichment of custard formulations with plant-derived ingredients significantly influences color characteristics after cooking.

#### 3.2. Sensory Evaluation

**Figure 1:**
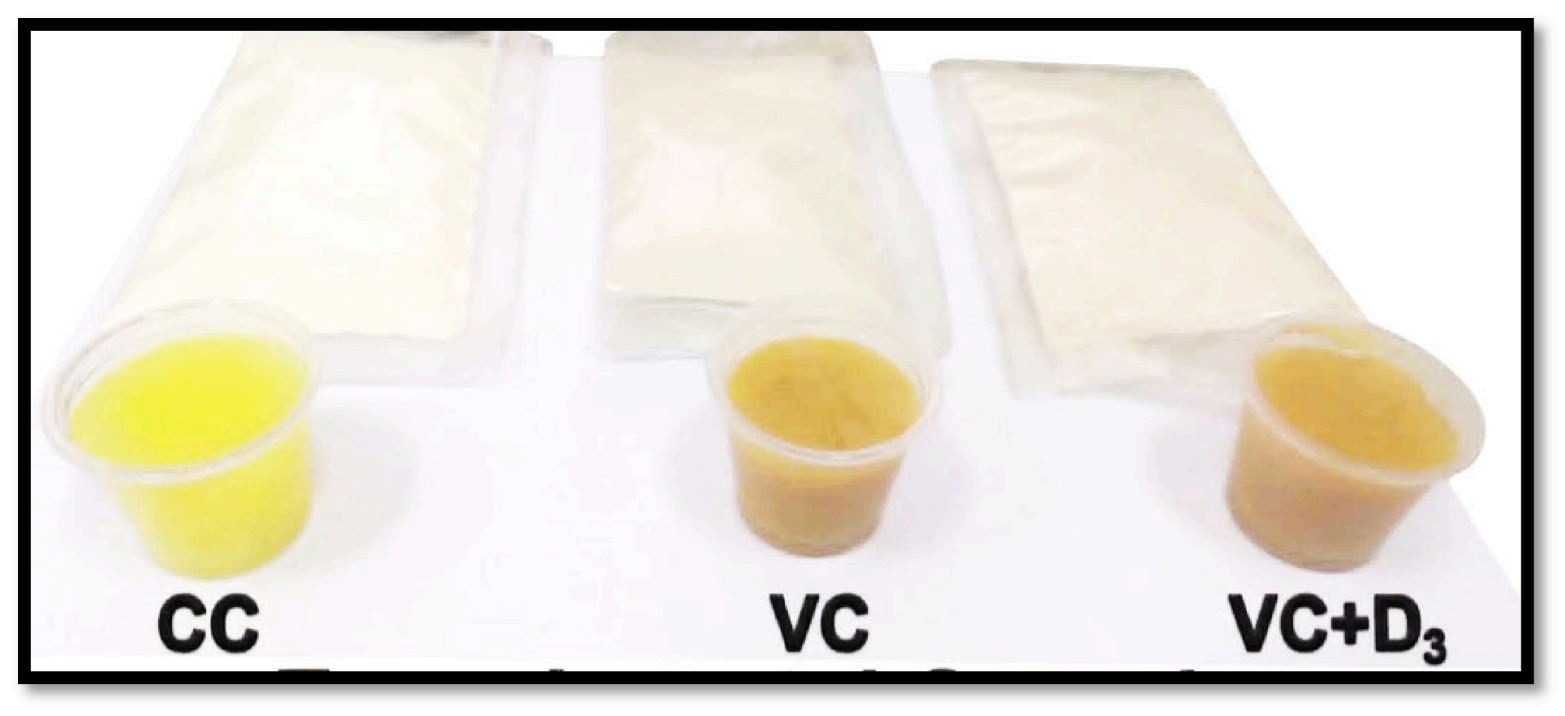
Visual appearance of different custard samples.

Sensory evaluation reflects consumers’ perception and acceptability of food products based on attributes such as flavor, texture, and overall palatability. As shown in Table 4 and Figure 1, the vegan custard formulations (VC and VC+D_3_) achieved significantly higher scores for flavor, mouth feel, taste, and overall acceptability compared with the commercial custard sample. These improvements may be attributed to the incorporation of dried banana powder, which contains volatile aroma compounds such as 3-methylbutyl ester, 3-methylbutyl acetate, and 3-methylbutanoic acid derivatives that contribute to enhanced flavor and consumer acceptability [39].

#### 3.3. Dispersibility, Packed Bulk Density and Solubility of Custard Powder Samples

Table 5 shows that dispersibility values ranged from 63.27% to 69.07%. Dispersibility reflects the ability of a flour sample to reconstitute rapidly in water. The results indicated a decrease in dispersibility in the VC+D_3_ sample (63.27%) compared with the commercial custard (69.07%) and VC sample (66.27%). Lower dispersibility values were associated with increased lump formation in the prepared gruels. These findings are consistent with those reported by Akinwale et al. [40], who observed that lower powder dispersibility is associated with a higher tendency for lump formation.

Bulk density (BD) is an important physical property influencing the packaging, handling, and transportation efficiency of powdered food products. In the present study, BD values ranged from 0.45 to 0.73 g/mL among the investigated custard samples. As shown in Table 5, the VC+D_3_ sample exhibited the lowest bulk density value (0.45 g/mL), compared with the commercial custard (0.73 g/mL) and VC sample (0.55 g/mL). This reduction in bulk density may be associated with modifications in particle structure and increased lipophilic interactions resulting from the incorporation of vitamin D_3_ and dried banana powder [41].

Solubility values increased with increasing temperature in all custard samples. In addition, the vegan custard formulations (VC and VC+D_3_) exhibited higher solubility percentages than the commercial custard sample. This behavior may be attributed to the relatively higher protein content of the vegan formulations due to the incorporation of baobab flour, millet flour, and banana powder, which are recognized as sources of proteins and functional compounds [13,14,15,42]. Protein solubility is strongly influenced by pH and temperature conditions. When proteins are displaced from their isoelectric point under acidic or alkaline conditions, protein–water interactions become more pronounced, leading to enhanced solubility. Furthermore, moderate heating temperatures (40–50 °C) generally increase protein solubility, whereas excessive heating may induce protein denaturation after prolonged exposure [43].

#### 3.4. Texture Profile of Cooked Custard Samples

Texture profile analysis (TPA) is widely used in the food industry to evaluate the textural properties of food products because it provides valuable information related to sensory quality and consumer perception [44]. Instrumental TPA has been extensively applied to characterize the texture of protein-based gels and dessert products, including gellan–gelatin gels, gelatin desserts, and custard formulations [45].

The TPA parameters of the investigated custard samples are presented in Table 6. Hardness, defined as the maximum force recorded during the first compression cycle, reflects the strength of the gel structure under compression. The commercial custard exhibited the lowest hardness value (9.17 N), whereas the VC and VC+D_3_ samples showed higher values of 10.07 and 10.95 N, respectively. These findings are consistent with those reported by Chandra and Shamasundar [46].

Cohesiveness represents the internal bonding strength of the food matrix and indicates the extent to which a product can withstand deformation before rupture [47]. The VC+D_3_ sample exhibited the highest cohesiveness value (0.56), followed by VC (0.48), while the commercial custard showed the lowest value (0.44). This increase in cohesiveness suggests the formation of a more integrated and stable gel network in the vegan custard formulations, particularly in the vitamin-D_3_-fortified sample.

Springiness, which describes the ability of a sample to recover its original shape after deformation, was significantly higher in the commercial custard compared with the vegan custard samples (VC and VC+D_3_). In contrast, gumminess, defined as the energy required to disintegrate a semi-solid food product, was significantly higher in the VC+D_3_ sample (6.30) than in VC (5.20) and commercial custard (4.80). This behavior likely reflects the combined effects of increased hardness and cohesiveness in the fortified vegan custard formulation.

#### 3.5. Nutritive Values and Antioxidant Properties of Investigated Products

As shown in Table 7 and Table 8, the innovative custard formulations (VC and VC+D_3_) exhibited significantly higher contents of moisture, fat, protein, fiber, energy, calcium, and phosphorous compared with the commercial custard (CC). In contrast, available carbohydrate content was significantly lower in the vegan formulations. Vitamins C, total phenolics (mg GAE/g), and DPPH were absent from the commercial custard product, whereas both plant-based custard formulations showed measurable levels of these bioactive compounds. Vitamin D was detected only in the fortified formulation (VC+D_3_). These observations are attributable to the fact that the plant-based custards contained baobab flour, millet flour, and banana powder, all of which are sources of proteins, vitamins, minerals, and bioactive compounds.

The literature confirms this fact. In their respective studies, Dossa et al. [14] and Silva et al. [48] have shown that baobab flour is a good functional ingredient owing to the presence of polyphenols, flavonoids, carbohydrates, lipids, and mineral contents, including potassium, calcium, magnesium, iron, sodium, and zinc.

Similarly, Hassan et al. [49] found out that pearl millet grain (Pennisetum americanum, var. Shandawel-1) contains high amounts of protein, fiber, fat, calcium, phosphorus, antioxidant activity, and total phenolic compounds. It can be said, thus, that the improvement in nutritional quality and antioxidants in this present study are likely associated with the combined contribution of these plant-based ingredients.

The obtained results further showed that a 100 g serving of the innovative plant-based custard formulations could help fulfill the recommended dietary allowance (RDA) set forth by the Institute of Medicine, Food and Nutrition Board [50]. As far as calcium is concerned, it was found that its contribution was from 4.07% to 4.40% in the case of children (4–8 years old), adults, and pregnant or lactating women (ages 19–70 years). However, when it comes to adolescents (9–18 years old) and pregnant or lactating females aged 14–18 years, its contribution ranged from 3.12% to 3.30%. Moreover, 100 g of the vitamin-D_3_-fortified custard powder can provide 333.3% of the RDA for vitamin D_3_, meeting the needs of children, teenagers, adults, as well as pregnant and lactating women.

#### 3.6. Calcium Bioaccessibility of Custard Samples

**Figure 2:**
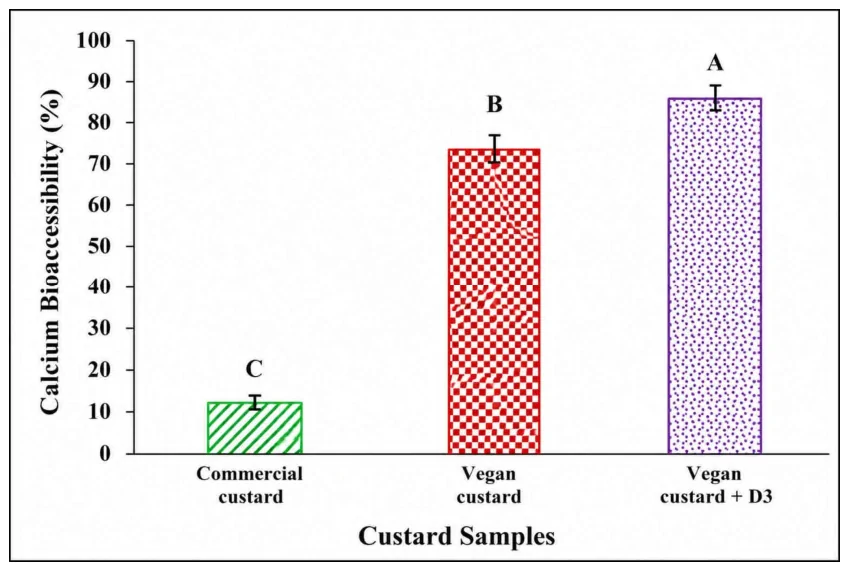
Comparative calcium bioaccessibility of custard samples. Different letters indicate significant differences.

The results of calcium bioaccessibility in commercial custard, vegan custard (VC), and vitamin-D_3_-fortified vegan custard (VC+D_3_) are presented in Figure 2. Following in vitro digestion, the highest calcium bioaccessibility was observed in the VC+D_3_ sample (85.3%), followed by the VC sample (71.3%). In contrast, the commercial custard exhibited the lowest calcium bioaccessibility value (12.3%). In this context, the improved bioaccessibility of calcium of plant-based custard formulations could be attributed to the higher calcium levels, as illustrated in Table 7, along with the presence of vitamin C and the characteristics of the food matrix, which facilitated calcium release during the simulated gastrointestinal digestion process. In addition, vitamin C may have contributed to improving mineral solubility during digestion, thereby enhancing calcium bioaccessibility [51]. It should be noted that the in vitro digestion model evaluates calcium bioaccessibility, defined as the fraction of calcium released from the food matrix during simulated gastrointestinal digestion, rather than intestinal calcium absorption. Moreover, these findings should be interpreted as relative indicators of calcium bioaccessibility obtained under in vitro experimental conditions rather than direct measures of in vivo calcium absorption.

#### 3.7. Effect of Vitamin-D_3_-Fortified Vegan Custard on Body Weight Gain, Feed Intake, Feed Efficiency Ratio (FER), Daily Nutrient Intakes and Femur Weight

As illustrated in Table 9, the consumption of the vegan custard formulations (Groups II and III) resulted in significant increases in body weight gain percentage (BWG%), feed intake (FI), feed efficiency ratio (FER), and femur weight as compared to the positive control group (low-calcium diet) and Group I (commercial custard formulation). Consequently, these animals consumed greater amounts of energy and calcium, while protein intake showed a slight but non-significant increase. These intake values were calculated based on the daily feed intake and the nutrient composition of each diet.

The observed increase in BWG% and femur weight may be attributed to the high nutritional content of the plant-based custard formulations that included increased amounts of proteins, minerals and bioactive components. Furthermore, the noted increase in BWG% may be explained by the noted increase in FI and FER values recorded for the treated groups, thus implying high efficiency in the utilization of the consumed food.

Besides, the incorporation of banana powder in the preparation of the plant-based custard formulations (VC and VC+D_3_) may be considered a key factor contributing to increased feed palatability due to the unique aroma characteristics that stimulated voluntary feed consumption in the treated groups. The mentioned effect was supported by the noted increase in FI value for Groups II and III. One-way ANOVA reveals a highly significant variation in vitamin D_3_ intake across the five experimental groups. Group III exhibits a markedly higher vitamin D_3_ intake (mean of 1.260) compared to all other groups, and negative control, positive control, Group I and Group II show relatively lower intake values ranging between 0.547 and 0.667, with no statistically significant differences among them.

#### 3.8. Effect of Vitamin-D_3_-Fortified Vegan Custard on Some Biochemical Investigations and Bone Tissue Improvement

Table 10 illustrates the changes in serum biochemical parameters and femur calcium content in rats fed a low-calcium diet alone (positive control) or supplemented with 10% commercial custard (Group I), 10% vegan custard (Group II), or 10%-vitamin-D_3_-fortified vegan custard (Group III), compared with the negative control group fed a normal-calcium diet.

The results demonstrated that feeding rats a low-calcium diet alone or supplemented with 10% commercial custard resulted in significant reductions in serum calcium, calcitonin, and femur calcium content, accompanied by significant increases in serum parathyroid hormone (PTH), alkaline phosphatase (ALP), and tartrate-resistant acid phosphatase (TRACP) levels compared with the other treated groups.

**Figure 3:**
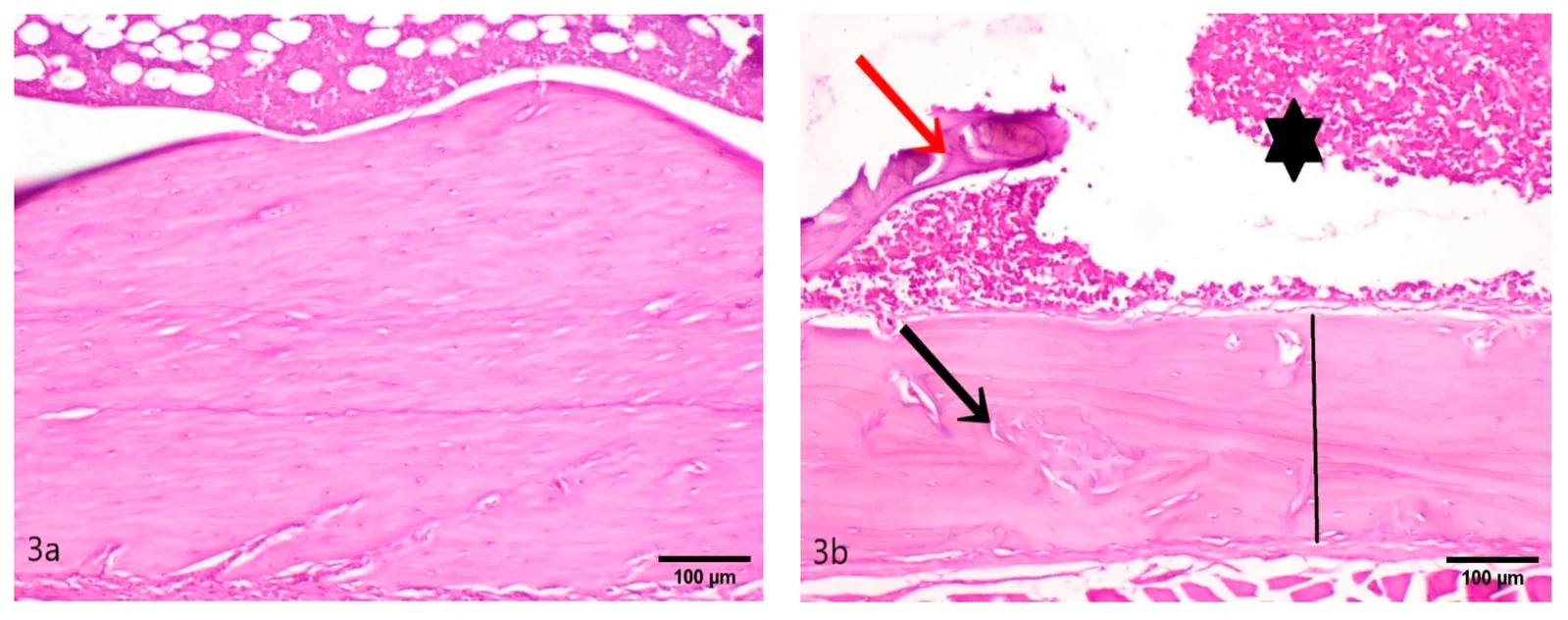

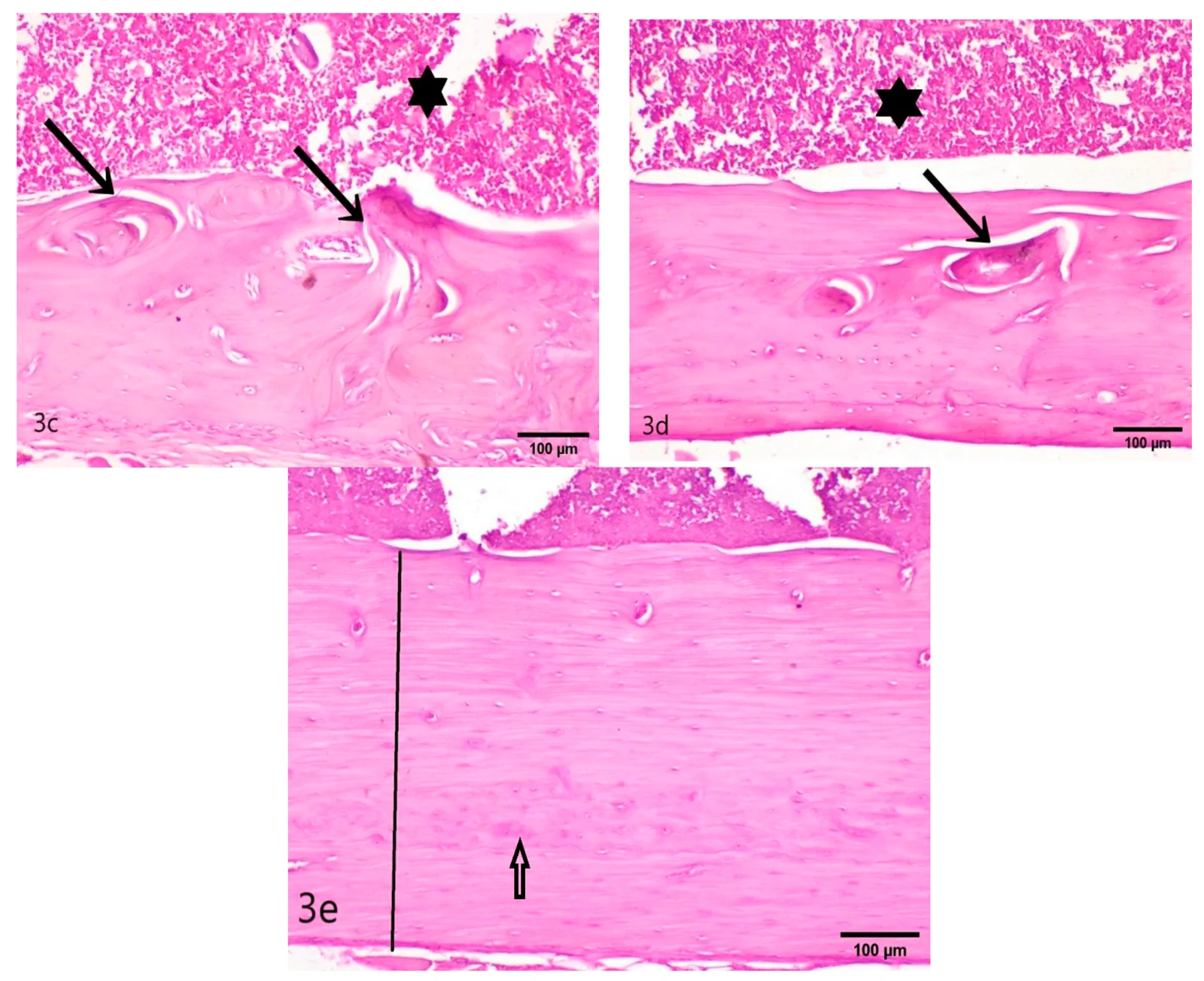
Histopathological alterations among experimental groups (H & E ×100, scale bar, 100 µm). Representative sections from femurs of 7 rats per group, 5 fields analyzed per section. (**a**) Section in a femur of rat from negative control group which fed on normal-calcium diet showing no histopathological lesions with normal bone cortex. (**b**) Section in a femur of rat from positive control group which fed on low-calcium diet showing thin cortical bone (line) with cracks and fissures (black arrow), thin bony trabeculae (red arrow) and dilated bone marrow cavity (asterisk). (**c**) Section in a femur of rat from group I which fed on low-calcium diet containing 10% commercial custard showing cracks and fissures in the cortical bone (black arrow) and dilated bone marrow cavity (asterisk). (**d**) Section in a femur of rat from Group II which fed on low-calcium diet containing 10% vegan custard showing moderate improvement with some fissures in the cortical bone (black arrow) and dilated bone marrow cavity (asterisk). (**e**) Section in a femur in a rat from Group III which fed on low-calcium diet containing 10%-D_3_-fortified vegan custard showing normal cortical bone (line) associated with proliferation of osteoblasts (arrow).

Microscopic examination of femoral tissues from the negative control group fed a normal-calcium diet for 30 days revealed no histopathological alterations. The examined sections showed normal cortical bone with gradual transformation of the fibrous callus occupying the bone defect into osteoid tissue, which subsequently developed into mature cortical bone (Figure 3a).

In contrast, femoral tissues from the positive control group fed the low-calcium diet exhibited marked histopathological alterations characterized by thinning of the cortical bone associated with cracks and fissures, thin bony trabeculae, and dilation of the bone marrow cavity (Figure 3b). Similarly, rats in Group I fed the low-calcium diet supplemented with 10% commercial custard showed severe histopathological changes with no obvious improvement compared with the positive control group (Figure 3c).

Conversely, femoral tissues from Group II rats fed the low-calcium diet supplemented with 10% vegan custard demonstrated moderate histological improvement. Some examined sections showed nearly normal cortical bone architecture, whereas others exhibited relatively thick cortical bone with only a few fine fissures (Figure 3d). The most pronounced improvement was observed in Group III rats fed the low-calcium diet supplemented with 10%-vitamin-D_3_-fortified vegan custard. These samples showed nearly normal cortical bone associated with osteoblastic proliferation, although a few fine fissures were still observed in some sections (Figure 3e).

The observed histological improvements in the VC+D_3_ group may be related to the physiological role of vitamin D in bone metabolism and restoration. Vitamin D plays an important role in the synthesis of bone matrix proteins and is involved in bone formation as it contributed to osteoblast differentiation and activity. Furthermore, osteocalcin and alkaline phosphatase, which are crucial for calcium transport, mineralization, and calcium deposition inside bone tissue, are expressed more when vitamin D is present [52]. By preserving the equilibrium between osteoclastic bone resorption and bone production, vitamin D also helps control bone remodeling. Adequate vitamin D levels have been linked to better bone healing and increased bone mineralization, especially in the early phases of bone repair [53]. Insufficient vitamin D levels might hinder bone growth and slow down the healing process. Additionally, osteoblast proliferation and differentiation, bone turnover, bone mineral density, and bone strength have all been shown to be enhanced by vitamin D [54,55,56,57]. Reid et al. [58] reported that vitamin D supplementation improved bone mineral density and activated bone healing in osteoporotic conditions.

Histomorphometric evaluations confirmed the histological findings as osteoblast count and cortical bone thickness were significantly diminished in the positive control group and Group I when compared with the negative control group. On the other hand, Group III revealed a significant improvement in osteoblast count and cortical bone thickness when compared to Group I (Table 11). 

Bone repair is a highly complex physiological process regulated by several hormones and enzymes involved in maintaining the balance between bone formation and bone resorption. Parathyroid hormone (PTH), one of these regulating factors, is crucial for maintaining calcium homeostasis because it promotes osteoclastic bone resorption, which raises calcium levels in the blood. On the other hand, it has also been noted that occasional PTH stimulation encourages bone growth [59]. On the other hand, the thyroid gland secretes calcitonin, which reduces bone resorption and inhibits osteoclast activity, counteracting the effects of PTH and helping to maintain bone mass and density [60].

Alkaline phosphatase (ALP), an enzyme produced by osteoblasts, is frequently utilized as a biomarker of osteoblastic activity and bone repair since it is closely linked to the processes of bone formation and mineralization [61]. On the other hand, osteoclast activity is tightly linked to tartrate-resistant acid phosphatase (TRACP), and elevated TRACP levels are typically thought to be a sign of accelerated bone turnover and resorption [62]. When taken as a whole, these biochemical indicators offer crucial insights into the dynamic equilibrium between bone resorption and creation during bone remodeling.

In addition to its impact on osteoclast activation, PTH indirectly regulates osteoblastic activity through the RANKL/OPG signaling pathway, which affects the processes of bone production and resorption [63]. In addition to its inhibitory effects on osteoclast activity, calcitonin has also been shown to affect bone mineralization and osteoblast viability [60].

ALP plays a pivotal role in bone mineralization through hydrolysis of inorganic pyrophosphate, a natural inhibitor of mineral deposition [64]. Therefore, elevated serum ALP activity is frequently regarded as a sign of increased osteoblastic activity and bone production. On the other hand, TRACP, especially the TRACP5b isoform, is thought to be a trustworthy indicator of bone resorption and osteoclastic activity. Increased bone turnover and active bone resorption processes are typically indicated by elevated TRACP levels [65].

The positive benefits of vitamin D supplementation on bone metabolism and repair have been shown in a number of prior studies. According to Muresan et al. [66], in experimental animal models, dietary vitamin D supplementation improved bone mineral density and bone repair. Similarly, in rat bone tissue, Skalny et al. [67] showed that vitamin D treatment greatly increased calcium deposition and bone mineralization. In osteopenic rats, Sundar et al. [57] also found enhanced calcium deposition and better bone improvement after vitamin D administration. Furthermore, vitamin-D-enriched diets increased bone density and mineral content in osteoporotic rat models, according to Osiak-Wicha et al. [68]. Numerous experimental studies have found similar results demonstrating the beneficial effect of vitamin D in bone mineralization and bone formation [66,69].

Regarding histological and histomorphometric results, earlier studies using oral or injectable vitamin D_3_ therapy in rats showed improved bone mineral content and increased new bone growth. Higher dosages of vitamin D_3_ were found to improve bone repair in rats with calvarial defect models, with newly produced bone tissue closely approximating normal bone architecture [70]. Similarly, vitamin D_3_ administration improved trabecular growth and decreased bone marrow gaps in rat mandibles, indicating better bone mineralization and quality, according to Muresan et al. [66]. Further demonstrating the advantageous effect of vitamin D in bone regeneration processes, intravenous calcitriol administration was linked to enhanced osseointegration and bone healing in osteoporotic mice.

Based on the serum biochemical results observed in the vitamin-D_3_-fortified vegan custard group, reductions in PTH, ALP, and TRACP levels were accompanied by increased calcitonin, serum calcium, and femur calcium levels compared with positive control and Group I. These findings suggest enhanced bone formation and reduced bone resorption, which were further supported by the histological observations showing thicker trabeculae and improved bone repair in the VC+D_3_ group. Collectively, these findings highlight the potential role of vitamin D_3_ fortification in improving bone physiology and calcium metabolism under low-calcium experimental conditions [71,72].

### 4. Conclusions

Our innovative vegan custard products, especially vitamin-D3-fortified vegan custard, showed a high nutritional value, calcium bioaccessibility and technological quality when prepared under their optimized preparation conditions. They contained high proportions of the recommended dietary allowances and can be considered functional foods for individuals with dairy allergies, lactose intolerance, and other special dietary requirements. A 100 g serving of the vitamin-D_3_-fortified vegan custard powder provided 333.3% of the recommended dietary allowance (RDA) of vitamin D. Based on the vitamin D content of the formulated product, a 30 g serving may substantially contribute to daily vitamin D requirements under the experimental formulation conditions. Differences in solid concentration resulting from the optimized preparation conditions may have influenced some technological properties. Furthermore, feeding rats with both innovative vegan custard formulations, particularly the vitamin-D_3_-fortified variant, had a significant positive impact on abnormalities induced by a low-calcium diet. Improvements were observed in body weight, certain serum biochemical markers, including calcium, parathyroid hormone (PTH), calcitonin (determined using rat PTH and calcitonin assays), tartrate-resistant acid phosphatase (TRACP), and alkaline phosphatase (ALP). Moreover, femur calcium content, histological and histomorphometric quantitative analysis of bone tissue showed significant improvements in femoral bone repair in the VC+D_3_ group.

## Figures and Tables

**Table 1:** The ingredients of normal-calcium diet and low-calcium diet (Diet composition (g/100 g)).

Ingredient	Normal-Calcium Diet	Low-Calcium Diet
Casein (g)	24.5	24.5
Corn oil (g)	6.0	6.0
Corn starch (g)	37.5	39.87
Potato starch (g)	1.0	1.0
Sucrose (g)	15.5	15.5
Cellulose (g)	5.0	5.0
Vitamin mix ^a^ (g)	1.0	1.0
Mineral mix ^b^ (g)	7.0	7.0
CaCO_3_ (g)	2.5	0.13

^a^ Vitamin mix contains (per 100 g) VA 1517 IU, VD3 250 IU, VE 7.0 mg, VB1 1.7 mg, VB2 1.3 mg, VB6 1.2 mg, VB12 3.4 mg, VC 19 mg, pantothenic acid 3.7 mg, niacin 16.7 mg, folic acid 0.2 mg, choline 195 mg, biotin 48.4 mg, nicotinic acid 549 mg. ^b^ Mineral mix contains (mg/100 g) KH_2_PO_4_ 1730, NaCl 600, FeC_6_H_5_O_7_·5H_2_O 190, 5ZnO·2CO_2_·4H_2_O 6, CuSO_4_·5H_2_O 1.26, CoCl_2_·6H_2_O 0.4, Ca(IO_3_)_2_ 1.54, MnSO_4_·4H_2_O 15.4, MgSO_4_·7H_2_O 800, Na_2_HPO_4_ 1240.

**Table 2:** Color parameter for custard powder (mean ± SE).

Samples	L*	a*	b*	Chrom*	Hueº
Commercial Custard (CC)	99.59 ± 0.01 ^A^	−2.81 ± 0.01 ^C^	9.34 ± 0.01 ^B^	9.76 ± 0.01 ^B^	106.77 ± 0.01 ^A^
Vegan Custard (VC)	89.77 ± 0.01 ^B^	4.03 ± 0.00 ^B^	12.71 ± 0.01 ^A^	13.33 ± 0.01 ^A^	72.43 ± 0.01 ^B^
Vegan Custard + D_3_ (VC+D_3_)	89.83 ± 0.01 ^B^	4.85 ± 0.01 ^A^	12.69 ± 0.01 ^A^	13.40 ± 0.01 ^A^	68.78 ± 0.01 ^C^

There is no significant difference between any two means within the same row having the same superscript letter.

**Table 3:** Color parameter for cooked custard (mean ± SE).

Samples	L*	a*	b*	Chrom*	Hueº
Commercial Custard (CC)	66.46 ± 0.01 ^A^	−7.47 ± 0.01 ^C^	24.11 ± 0.01 ^B^	25.26 ± 0.01 ^C^	107.22 ± 0.01 ^A^
Vegan Custard (VC)	55.81 ± 0.01 ^B^	−3.61 ± 0.01 ^B^	66.16 ± 0.01 ^A^	66.26 ± 0.01 ^A^	93.12 ± 0.01 ^B^
Vegan Custard + D_3_ (VC+D_3_)	55.13 ± 0.01 ^C^	1.61 ± 0.01 ^A^	66.18 ± 0.01 ^A^	66.18 ± 0.01 ^B^	88.62 ± 0.01 ^C^

There is no significant difference between any two means within the same row having the same superscript letter.

**Table 4:** Sensory evaluation of cooked custard samples (mean ± SE).

Samples	Appearance	Flavor	Mouth Feel	Taste	Overall Acceptability
Commercial Custard (CC)	8.46 ± 0.02 ^A^	6.44 ± 0.01 ^B^	7.82 ± 0.04 ^B^	6.62 ± 0.01 ^B^	7.73 ± 0.01 ^C^
Vegan Custard (VC)	8.43 ± 0.02 ^A^	8.95 ± 0.02 ^A^	8.91 ± 0.01 ^A^	8.92 ± 0.01 ^A^	8.94 ± 0.01 ^A^
Vegan Custard (VC+D_3_)	8.47 ± 0.03 ^A^	8.97 ± 0.02 ^A^	8.92 ± 0.01 ^A^	8.94 ± 0.02 ^A^	8.96 ± 0.01 ^A^

There is no significant difference between any two means, within the same row have the same superscript letter.

**Table 5:** Dispersibility, packed bulk density and solubility of custard powder samples (mean ± SE).

Parameter Tests		Samples		
		Commercial Custard (CC)	Vegan Custard (VC)	Vegan Custard + D_3_ (VC+D_3_)
Solubility (%) at	40 °C	0.12 ± 0.01 ^B^	0.15 ± 0.01 ^A^	0.15 ± 0.01 ^A^
	50 °C	0.29 ± 0.01 ^B^	0.34 ± 0.01 ^A^	0.35 ± 0.01^A^
	60 °C	0.40 ± 0.01 ^B^	0.45 ± 0.01 ^A^	0.46 ± 0.01 ^A^
Dispersibility (%)		69.07 ± 0.09 ^A^	66.27 ± 0.15 ^B^	63.27 ± 0.03 ^C^
Bulk Density (g/mL)		0.73 ± 0.01 ^A^	0.55 ± 0.01 ^B^	0.45 ± 0.01 ^C^

There is no significant difference between any two means within the same row having the same superscript letter. Commercial custard (CC); Vegan custard (VC); Vegan+D_3_ (VC+D_3_).

**Table 6:** Texture profile of cooked custard samples (mean ± SE).

Parameter Tests	Samples		
	Commercial Custard (CC)	Vegan Custard (VC)	Vegan Custard + D_3_ (VC+D_3_)
Hardness (N)	9.17 ± 0.07 ^C^	10.07 ± 0.04 ^B^	10.95 ± 0.03 ^A^
Cohesiveness (-)	0.44 ± 0.01 ^C^	0.48 ± 0.00 ^B^	0.56 ± 0.01 ^A^
Springiness (mm)	19.17 ± 0.09 ^A^	18.50 ± 0.06 ^C^	18.73 ± 0.04 ^B^
Gumminess (-)	4.80 ± 0.06 ^C^	5.20 ± 0.06 ^B^	6.30 ± 0.12 ^A^

There is no significant difference between any two means within the same row having the same superscript letter.

**Table 7:** Nutritional values of custard powder samples (mean ±SE).

Parameters	Commercial Custard (CC)	Vegan Custard (VC)	Vegan Custard + D_3_ (VC+D_3_)
Moisture (%)	5.60 ± 0.06 ^B^	6.73 ± 0.09 ^A^	6.73 ± 0.09 ^A^
Crude fat (%)	0.75 ± 0.01 ^B^	4.47 ± 0.09 ^A^	4.47 ± 0.09 ^A^
Crude protein (%)	1.01 ± 0.21 ^B^	4.20 ± 0.12 ^A^	4.20 ± 0.12 ^A^
Crude fiber (%)	0.13 ± 0.01 ^B^	1.05 ± 0.01 ^A^	1.05 ± 0.01 ^A^
Available carbohydrates (g/100 g, dry basis)	97.82 ± 0.13 ^A^	89.2 ± 0.04 ^B^	89.2 ± 0.04 ^B^
Energy (kcal/100 g)	402.07 ± 0.79 ^B^	413.83 ± 1.65 ^A^	413.83 ± 1.65 ^A^
Calcium (mg/100 g)	1.93 ± 0.24 ^B^	40.67 ± 0.67 ^A^	40.67 ± 0.67 ^A^
Phosphorus (mg/100 g)	11.00 ± 0.58 ^B^	33.67 ± 2.19 ^A^	33.67 ± 2.19 ^A^
VD (µg/100 g)	-	-	50.05. ± 0.04 ^A^
Vitamin C (mg/100 g, as ascorbic acid)	-	38.00 ± 0.58 ^A^	38.00 ± 0.58 ^A^

There is no significant difference between any two means within the same row having the same superscript letter.

**Table 8:** Total phenolic compounds and DPPH of custard powder samples (mean ± SE).

Component	Commercial Custard (CC)	Vegan Custard (VC)	Vegan Custard + D_3_ (VC+D_3_)
Total phenolic compounds (mg GAE/g)	0.93 ± 0.22 ^B^	4.6 ± 0.08 ^A^	4.6 ± 0.08 ^A^
DPPH (%)	1.17 ± 0.38 ^B^	7.11 ± 0.04 ^A^	7.11 ± 0.04 ^A^

There is no significant difference between any two means within the same row having the same superscript letter.

**Table 9:** Body weight Gain %, Feed intake, Feed efficiency ratio (FER), Daily nutrient intakes and Femur weight (mean ± SE).

Parameter	Negative Control	Positive Control	Group I	Group II	Group III
Body weight Gain BWG (%)	28.67 ± 2.40 ^B^	14.47 ± 2.86 ^C^	19.00 ± 0.58 ^C^	45.67 ± 0.67 ^A^	50.00 ± 0.58 ^A^
Feed intake (FI, g/rat/day)	9.95 ± 0.74 ^C^	9.21 ± 0.59 ^D^	8.79 ± 082 ^E^	10.67 ± 0.082 ^B^	11.15 ± 0.45 ^A^
Protein intake (g/rat/day)	2.18 ± 0.7 ^A^	2.02 ± 0.5 ^A^	1.77 ± 08 ^A^	1.79 ± 0.08 ^A^	2.22 ± 0.45 ^A^
Energy intake (Kcal/rat/day)	36.5 ± 0.58 ^C^	33.74 ± 0.48 ^D^	32.50 ± 049 ^D^	39.61 ± 0.55 ^B^	41.40 ± 0.65 ^A^
Calcium intake (mg/rat/day)	99.33 ± 0.44 ^A^	4.56 ± 0.14 ^C^	4.08 ± 08 ^C^	5.51 ± 0.25 ^B^	5.88 ± 0.35 ^B^
Vitamin D_3_ (μg/rat//day)	0.622 ± 0.019 ^B^	0.576 ± 0.021 ^B^	0.547 ± 0.020 ^B^	0.667 ± 0.054 ^B^	1.260 ± 0.087 ^A^
Feed efficiency ratio (FER)	0.060 ± 0.01 ^B^	0.029 ± 0.00 ^D^	0.0437 ± 0.00 ^C^	0.0867 ± 0.00 ^A^	0.092 ± 0.00 ^A^
Femur weight (mg)	83.67 ± 0.88 ^A^	76.00 ± 0.58 ^C^	75.00 ± 1.15 ^C^	78.33 ± 0.33 ^B^	80.33 ± 0.33 ^B^

There is no significant difference between any two means within the same row having the same superscript letter. Feed intake was recorded on a per-rat basis, and each rat was considered the experimental unit for feed intake analysis. Daily nutrient intakes (protein, energy, calcium, and vitamin D_3_) were calculated from the mean daily feed intake and the nutrient composition of the corresponding diet.

**Table 10:** Biochemical Changes during experimental study (mean ± SE).

Parameter	Negative Control	Positive Control	Group I	Group II	Group III
Serum calcium (mg/100 mL)	12.53 ± 0.19 ^A^	9.97 ± 0.09 ^B^	10.13 ± 0.12 ^B^	11.83 ± 0.03 ^A^	12.70 ± 0.60 ^A^
Femur calcium content (mg/100 g of body weight)	29.67 ± 0.33 ^A^	18.00 ± 0.58 ^D^	17.33 ± 0.67 ^D^	23.33 ± 0.88 ^C^	26.33 ± 0.67 ^B^
PTH (pg/mL)	13.90 ± 0.06 ^D^	52.00 ± 0.06 ^A^	51.90 ± 0.23 ^A^	21.20 ± 0.72 ^B^	18.17 ± 0.44 ^C^
Calcitonin (pg/mL)	64.43 ± 0.3 ^A^	54.43 ± 0.30 ^C^	53.67 ± 0.88 ^C^	59.03 ± 0.55 ^B^	62.67 ± 0.88 ^A^
ALP (U\L)	451.00 ± 0.58 ^C^	561.00 ± 4.51 ^A^	569.67 ± 5.49 ^A^	494.67 ± 3.18 ^B^	457.67 ± 2.85 ^C^
TRACP (U\L)	74.67 ± 0.67 ^D^	87.40 ± 0.50 ^A^	88.07 ± 0.66 ^A^	80.33 ± 0.33 ^B^	78.67 ± 0.33 ^C^

There is no significant difference between any two means within the same row having the same superscript letter.

**Table 11:** Histomorphometric Changes during experimental study (mean ± SE).

Parameter	Negative Control	Positive Control	Group I	Group II	Group III
Osteoblastic count (OC)	15.6 ± 0.50 ^A^	7.2 ± 0.58 ^C^	7.4 ± 0.24 ^C^	11.2 ± 0.37 ^B^	14.8 ± 0.20 ^A^
Cortical bone thickness (CBT) (μm)	451.4 ± 1.28 ^A^	327.6 ± 1.69 ^D^	380.2 ± 2.33 ^C^	422.6 ± 0.92 ^B^	438.2 ± 0.58 ^A^

Values are expressed as Mean ± SE (*n* = 7 animals/group). Each value represents the average of five microscopic fields analyzed per animal. There is no significant difference between any two means within the same row having the same superscript letter.

## Data Availability

The original contributions presented in this study are included in the article. Further inquiries can be directed to the corresponding authors.

## References

[1] Salami K.O., Olorunlambe A.A., Adesina B.O., Akinwande F.F., Ahmed El-Imam A.M., Oyeyinka S.A. (2019). Physicochemical and sensory properties of corn starch custard soured with tamarind, soursop, and lime. Croat. J. Food Technol. Biotechnol. Nutr..

[2] Olorunsogo S.T., Adejumo B.A., Abubakar A. (2023). Development, optimization and evaluation of enriched custard powder from selected local food ingredients. Int. J. Food Eng. Technol..

[3] Alimi B.A., Workneh T.S., Oyeyinka S.A. (2017). Structural, rheological and in-vitro digestibility properties of composite corn-banana starch custard paste. LWT—Food Sci. Technol..

[4] Tuszczki E., Boakye F., Zielińska M., Dereń K., Bartosiewicz A., Oleksy T., Stolarczyk A. (2023). Vegan diet: Nutritional components, implementation, and effects on adults’ health. Front. Nutr..

[5] Selinger E., Neuenschwander M., Koller A., Gojda J., Kühn T., Schwingshackl L., Barbaresko J., Schlesinger S. (2023). Evidence of a vegan diet for health benefits and risks—An umbrella review of meta-analyses of observational and clinical studies. Crit. Rev. Food Sci. Nutr..

[6] Gallagher C.T., Hanley P., Lane K.E. (2022). Pattern analysis of vegan eating reveals healthy and unhealthy patterns within the vegan diet. Public Health Nutr..

[7] Pilz S., Marz W., Cashman K.D., Kiely M.E., Whiting S.J., Holick M.F., Grant W.B., Pludowski P., Hiligsmann M., Trummer C. (2018). Rationale and plan for vitamin D food fortification: A review and guidance paper. Front. Endocrinol..

[8] McCourt A.F., Mulrooney S.L., O’Neill G., O’rIordan E.D., O’sUllivan A.M. (2022). Postprandial 25(OH)D response varies according to the lipid composition of a vitamin D3 fortified dairy drink. Int. J. Food Sci. Nutr..

[9] Bikle D.D., Feingold K.R., Adler R.A., Ahmed S.F., Anawalt B., Blackman M.R., Chrousos G., Corpas E., de Herder W.W., Dhatariya K., Dungan K. (2025). Vitamin D: Production, Metabolism, and Mechanism of Action. Endotext [Internet].

[10] Essa H. (2024). Potential role of vitamin D in health and disease: A literature review. Egypt. J. Chem..

[11] Voiculescu V.M., Nelson Twakor A., Jerpelea N., Pantea Stoian A. (2025). Vitamin D: Beyond Traditional Roles-Insights into Its Biochemical Pathways and Physiological Impacts. Nutrients.

[12] Burns-Whitmore B., Froyen E.B., Isom K.A. (2024). Vitamin D and Calcium—An Overview, Review of Metabolism, and the Importance of Co-Supplementation. Dietetics.

[13] Nibhoria A., Kumar M., Arya R.K., Siroha A.K. (2024). Pearl millet for good health and nutrition—An overview. CABI Rev..

[14] Dossa S., Dragomir C., Plustea L., Dinulescu C., Cocan I., Negrea M., Berbecea A., Alexa E., Rivis A. (2023). Gluten-free cookies enriched with baobab flour (*Adansonia digitata* L.) and buckwheat flour (*Fagopyrum esculentum*). Appl. Sci..

[15] Kumari P., Gaur S.S., Tiwari R.K. (2023). Banana and its by-products: A comprehensive review on its nutritional composition and pharmacological benefits. eFood.

[16] Rahayu M.A., Hudi L. (2021). The Effect of Blanching Time and Sodium Metabisulfite Concentration on The Characteristics of Banana Flour (*Musa paradisiaca*). J. Trop. Food Agroindustrial Technol..

[17] Kamel R.M., El-Kholy M.M., Abdelmotaleb I.A. (2017). Drying of banana slices under vacuum-infrared heating system. Misr J. Agric. Eng..

[18] Nikooyeh B., Neyestani T.R., Zahedirad M., Mohammadi M., Hosseini S.H., Abdollahi Z., Salehi F., Mirzay Razaz J., Shariatzadeh N., Kalayi A. (2016). Vitamin D-fortified bread is as effective as supplement in improving vitamin D status: A randomized clinical trial. J. Clin. Endocrinol. Metab..

[19] Singh S., David J. (2017). Development of pudding with different levels of water chestnut (*Trapa bispinosa*) powder. Pharma Innov..

[20] Cano-Chauca M., Stringheta P.C., Sardagna L.D., Cal-Vidal J. Mango juice dehydration spray drying using different carriers and functional characterization. Proceedings of the 14th International Drying Symposium.

[21] Oladele A.K., Aina J.O. (2007). Chemical composition and functional properties of flour produced from two varieties of tiger nut (*Cyperus esculentus*). Afr. J. Biotechnol..

[22] Kulkarni K.D., Kulkarni D.N., Ingle U.M. (1991). Sorghum malt-based weaning formulations: Preparation, functional properties, and nutritive value. Food Nutr. Bull..

[23] Latimer G.W. (2012). Official Methods of Analysis of AOAC International.

[24] Fraser J.R., Holmes D.C. (1959). Proximate analysis of wheat flour carbohydrates. IV.—Analysis of wholemeal flour and some of its fractions. J. Sci. Food Agric..

[25] James C.S., James C.S. (1995). General food studies. Analytical Chemistry of Foods.

[26] Bošnir J., Bevardi M., Hećimović I., Budeč M., Juranović Cindrić I., Kober R., Jurak G., Lasić D., Brkić D., Racz A. (2024). Optimization of Sample Preparation Procedure for Determination of Fat-Soluble Vitamins in Milk and Infant Food by HPLC Technique. Processes.

[27] Badejo A.A., Duyilemi T.I., Falarunu A.J., Akande O.A. (2020). Inclusion of baobab (*Adansonia digitata* L.) fruit powder enhances the mineral composition and antioxidative potential of processed tigernut (*Cyperus esculentus*) beverages. Prev. Nutr. Food Sci..

[28] Jagtap U.B., Waghmare S.R., Lokhande V.H., Suprasanna P., Bapat V.A. (2011). Preparation and evaluation of antioxidant capacity of Jackfruit (*Artocarpus heterophyllus* Lam.) wine and its protective role against radiation induced DNA damage. Ind. Crop. Prod..

[29] Mustafa M.A., Ashry M., Salama H.H., Abdelhamid S.M., Hassan L.K., Abdel-Wahhab K.G. (2020). Ameliorative role of ashwagandha/probiotics fortified yogurt against AlCl3 toxicity in rats. Int. J. Dairy Sci..

[30] Suliburska J., Krejpcio Z. (2014). Evaluation of the content and bioaccessibility of iron, zinc, calcium and magnesium from groats, rice, leguminous grains and nuts. J. Food Sci. Technol..

[31] Chen H., Hayakawa D., Emura S., Ozawa Y., Okumura T., Shoumura S. (2002). Effect of low or high dietary calcium on the morphology of the rat femur. Histol. Histopathol..

[32] Chapman D.G., Castilla R., Compel J.A. (1959). Evaluation of protein in foods. I-A: Method for determination of protein efficiency ratio. Can. J. Biochem. Physiol..

[33] Wawrzyniak N., Gramza-Michałowska A., Kurzawa P., Kołodziejski P., Suliburska J. (2023). Calcium carbonate-enriched pumpkin affects calcium status in ovariectomized rats. J. Food Sci. Technol..

[34] Sanford H.S. (1954). Method for obtaining venous blood from orbital sinus of the rat or mouse. Science.

[35] Belfield A., Goldberg D.M. (1971). Normal ranges and diagnostic value of serum 5′nucleotidase and alkaline phosphatase activities in infancy. Arch. Dis. Child..

[36] Steel R.G.D., Torrie J.H., Dickey D.A. (1997). Principles and Procedures of Statistics: A Biometrical Approach.

[37] Zarroug Y., Terras D.S., Khemakhem M., Hamdaoui G.H., Hessini K., Allouch W., Dridi F., Hadjyahia N., El Felah M., Kharrat M. (2022). Physicochemical charac-terization, antioxidant activity and phenolic acids profile of Tunisian whole cereal grains. J. Food Saf. Hyg..

[38] Su F., Wu Y., Cao Y., Wang S. (2024). Differences in the Chromogenic Effect of Corn Starch and Potato Starch on Paprika Red Pigment and Structural Characterisation. Foods.

[39] Biernacka B., Dziki D., Różyło R., Gawlik-Dziki U. (2020). Banana Powder as an Additive to Common Wheat Pas-ta. Foods.

[40] Akinwale T.E., Niniola D.M., Abass A.B., Shittu T.A., Adebowale A.A., Awoyale W., Awonorin S.O., Adewuyi S., Eromosele C.O. (2017). Screening of some cassava starches for their potential applications in custard and salad cream productions. J. Food Meas. Charact..

[41] Alimi B.A., Sibomana M.S., Workneh T.S., Oke M.O. (2016). Some engineering properties of composite corn-banana custard flour. J. Food Process Eng..

[42] Olatoye K., Olusanya O., Olaniran A. (2021). The nutritional characteristics and acceptability of baobab (*Adansonia digitata* L.) pulp as nutrient concentrate substitute in custard powder. Potravin. Slovak J. Food Sci..

[43] Pelegrine D.H.G., Gasparetto C.A. (2005). Whey proteins solubility as function of temperature and pH. LTW-Food Sci. Technol..

[44] Burey P., Bhandari B.R., Rutgers R.P.G., Halley P.J., Torley P.J. (2009). Confectionery gels: A review on formulation, rheological and structural aspects. Int. J. Food Prop..

[45] Lau M., Tang J., Paulson A.T. (2000). Texture profile and turbidity of gellan/gelatin mixed gels. Food Res. Int..

[46] Chandra M.V., Shamasundar B.A. (2015). Texture profile analysis and functional properties of gelatin from the skin of three species of freshwater fish. Int. J. Food Prop..

[47] Radocaj O.F., Dimic E.B., Vujasinovic V.B. (2011). Optimization of the texture of fat-based spread containing hull-less pumpkin (*Cucurbita pepo* L.) seed press-cake. Acta Period. Technol..

[48] Silva M.L., Rita K., Bernardo M.A., Mesquita M.F., Pintão A.M., Moncada M. (2023). Adansonia digitata L. (Baobab) Bioactive Compounds, Biological Activities, and the Potential Effect on Glycemia: A Narrative Review. Nutrients.

[49] Hassan E.M., Fahmy H.A., Magdy S., Hassan M.I. (2020). Chemical composition, rheological, organoleptical and quality attributes of gluten-free fino bread. Egypt. J. Chem..

[50] Institute of Medicine, Food and Nutrition Board (2010). Dietary Reference Intakes for Calcium and Vitamin D.

[51] Abdelrahim D.N., Alamery H., Shawareb L.M. (2020). Ascorbic acid (vitamin C) and its effect on 18 minerals bioavaila-bility in human nutrition. Int. J. Sci. Acad. Res..

[52] Pilz S., Zittermann A., Trummer C., Theiler-Schwetz V., Lerchbaum E., Keppel M.H., Grübler M.R., März W., Pandis M. (2019). Vitamin D testing and treatment: A narrative review of current evidence. Endocr. Connect..

[53] Cashman K.D. (2020). Vitamin D Deficiency: Defining, Prevalence, Causes, and Strategies of Addressing. Calcif. Tissue Int..

[54] van Driel M., van Leeuwen J.P.T.M. (2023). Vitamin D and Bone: A Story of Endocrine and Auto/Paracrine Action in Osteoblasts. Nutrients.

[55] Lips P., Cashman K.D., Lamberg-Allardt C., Bischoff-Ferrari H.A., Obermayer-Pietsch B., Bianchi M.L., Stepan J., El-Hajj Fuleihan G., Bouillon R. (2019). Current vitamin D status in European and Middle East countries and strategies to prevent vitamin D deficiency: A position statement of the European Calcified Tissue Society. Eur. J. Endocrinol..

[56] Gatt T., Grech A., Arshad H. (2023). The Effect of Vitamin D Supplementation for Bone Healing in Fracture Patients: A Systematic Review. Adv. Orthop..

[57] Sundar R., Bhagavandas Rai A., Naveen Kumar J., Devang Divakar D. (2023). The role of Vitamin D as an adjunct for bone regeneration: A systematic review of literature. Saudi Dent. J..

[58] Reid I.R., Bolland M.J., Grey A. (2014). Effects of vitamin D supplements on bone mineral density: A systematic review and meta-analysis. Lancet.

[59] Marques M.R., Dias da Silva M.A., Manzi F.R., Cesar-Neto J.B., Francisco Nociti F.H., Barros S.B. (2005). Effect of intermittent PTH administration in the periodontitis-associated bone loss in ovariectomized rats. Arch. Oral Biol..

[60] Xie J., Guo J., Kanwal Z., Wu M., Lv X., Ibrahim N.A., Li P., Buabeid M.A., Arafa E.A., Sun Q. (2020). Calcitonin and Bone Physiology: In Vitro, In Vivo, and Clinical Investigations. Int. J. Endocrinol..

[61] Haarhaus M., Cianciolo G., Barbuto S., La Manna G., Gasperoni L., Tripepi G., Plebani M., Fusaro M., Magnusson P. (2022). Alkaline Phosphatase: An Old Friend as Treatment Target for Cardiovascular and Mineral Bone Disorders in Chronic Kidney Disease. Nutrients.

[62] Vohora D., Parveen B., Preedy V. (2016). Tartrate-Resistant Acid Phosphatase as a Biomarker of Bone Remodeling. Biomarkers in Bone Disease.

[63] Li J.Y., Yu M., Pal S., Tyagi A.M., Dar H., Adams J., Weitzmann M.N., Jones R.M., Pacifici R. (2020). Parathyroid hormone-dependent bone formation requires butyrate production by intestinal microbiota. J. Clin. Investig..

[64] Chawla V., Bundel P., Singh Y. (2025). ALP-Mimetic Cyclic Peptide Nanotubes: A Multifunctional Strategy for Osteo-genesis and Bone Regeneration. Biomacromolecules.

[65] Rathod B., Samvelyan H.J., Desai S., Bock L., Gustafsson N., Wu J., Ohlsson C., Magnusson P., Andersson G., Windahl S.H. (2025). Tartrate-resistant acid phosphatase augments the bone anabolic response to mechanical loading in male mice. JBMR Plus.

[66] Muresan G.C., Hedesiu M., Lucaciu O., Boca S., Petrescu N. (2022). Effect of Vitamin D on Bone Regeneration: A Review. Medicina.

[67] Skalny A.V., Aschner M., Tsatsakis A., Rocha J.B.T., Santamaria A., Spandidos D.A., Martins A.C., Lu R., Korobeinikova T.V., Chen W. (2024). Role of vitamins beyond vitamin D3 in bone health and osteoporosis (Review). Int. J. Mol. Med..

[68] Osiak-Wicha C., Muszyński S., Kras K., Tomaszewska E., Szymczyk B., Oczkowicz M., Świątkiewicz M., Wiącek D., Kamiński D., Arciszewski M.B. (2026). Effects of dietary vitamin D supplementation on bone microarchitecture, mineralization, and mechanical properties in Wistar rat animal model. Sci. Rep..

[69] Hacibektasoglu M.S., Balcioglu H.A., Uyanikgil Y., Balcioglu N.B. (2024). The effects of systemic vitamin D level on the healing of different graft materials: An experimental histological study. Appl. Sci..

[70] Abdel-Khalek R.R., Aly H.M., Omar S.S., El-Adawy M.M. (2020). Effect of different doses of Vitamin D3 supplementation on mandibular bone in rats. Alex. Dent. J..

[71] Wu S., Wang L., Zhang X., Cai L., Ke Q., Xu J. (2026). Bone turnover markers (β-CTX, PINP, ALP) in osteoporosis: Cor-relation with bone loss and fracture risk stratification. Front. Endocrinol..

[72] Topyürek D., Meier C., Kränzlin M.E. (2025). Der Stellenwert der Knochenumbauparameter im Management der Osteoporose [The importance of bone remodelling parameters in the management of osteoporosis]. Ther. Umschau. Rev. Ther..

